# Effectiveness of a Web-Based Multiple Tailored Smoking Cessation Program: A Randomized Controlled Trial Among Dutch Adult Smokers

**DOI:** 10.2196/jmir.1812

**Published:** 2012-06-11

**Authors:** Eline Suzanne Smit, Hein de Vries, Ciska Hoving

**Affiliations:** ^1^Department of Health PromotionMaastricht UniversityMaastrichtNetherlands; ^2^CAPHRI School for Public Health and Primary CareMaastricht UniversityMaastrichtNetherlands

**Keywords:** Smoking cessation, Web-based intervention, computer tailoring, iterative feedback, intervention, randomized controlled trial

## Abstract

**Background:**

Distributing a multiple computer-tailored smoking cessation intervention through the Internet has several advantages for both provider and receiver. Most important, a large audience of smokers can be reached while a highly individualized and personal form of feedback can be maintained. However, such a smoking cessation program has yet to be developed and implemented in the Netherlands.

**Objective:**

To investigate the effects of a Web-based multiple computer-tailored smoking cessation program on smoking cessation outcomes in a sample of Dutch adult smokers.

**Methods:**

Smokers were recruited from December 2009 to June 2010 by advertising our study in the mass media and on the Internet. Those interested and motivated to quit smoking within 6 months (N = 1123) were randomly assigned to either the experimental (n = 552) or control group (n = 571). Respondents in the experimental group received the fully automated Web-based smoking cessation program, while respondents in the control group received no intervention. After 6 weeks and after 6 months, we assessed the effect of the intervention on self-reported 24-hour point prevalence abstinence, 7-day point prevalence abstinence, and prolonged abstinence using logistic regression analyses.

**Results:**

Of the 1123 respondents, 449 (40.0%) completed the 6-week follow-up questionnaire and 291 (25.9%) completed the 6-month follow-up questionnaire. We used a negative scenario to replace missing values. That is, we considered respondents lost to follow-up to still be smoking. The computer-tailored program appeared to have significantly increased 24-hour point prevalence abstinence (odds ratio [OR] 1.85, 95% confidence interval [CI] 1.30–2.65), 7-day point prevalence abstinence (OR 2.17, 95% CI 1.44–3.27), and prolonged abstinence (OR 1.99, 95% CI 1.28–3.09) rates reported after 6 weeks. After 6 months, however, no intervention effects could be identified. Results from complete-case analyses were similar.

**Conclusions:**

The results presented suggest that the Web-based computer-tailored smoking cessation program had a significant effect on abstinence reported after a 6-week period. At the 6-month follow-up, however, no intervention effects could be identified. This might be explained by the replacement of missing values on the primary outcome measures due to attrition using a negative scenario. While results were similar when using a less conservative scenario (ie, complete-case analyses), the results should still be interpreted with caution. Further research should aim at identifying strategies that will prevent high attrition in the first place and, subsequently, to identify the best strategies for dealing with missing data when studies have high attrition rates.

**Trial Registration:**

Dutch Trial Register NTR1351; http://www.trialregister.nl/trialreg/admin/rctview.asp?TC=1351 (Archived by WebCite at http://www.webcitation.org/67egSTWrz)

## Introduction

Worldwide, the smoking of tobacco is the most preventable cause of illness and premature death [1]. Therefore, many interventions have been developed aimed at helping smokers to quit. One strategy that has shown both short- and long-term efficacy in changing smoking behavior is computer tailoring [2-7]. The content of a computer-tailored intervention is adapted to the specific characteristics of a particular individual. This has been shown to attract and keep an individual’s attention [8,9], resulting in more thorough processing of information [10]. A single tailored feedback message has already proven to be effective in promoting abstinence from smoking [11], but when tailored information is provided on multiple occasions the impact of the intervention can be increased even more [2,12]. In addition, a recent meta-analysis found that dynamically tailored interventions (ie, iterative assessments and feedback) resulted in greater changes in health behavior than statically tailored interventions [13]. As this effect could not be explained solely by the increased number of overall contacts that dynamic tailoring necessitates [13], a computer-tailoring approach may be warranted that consists not only of multiple feedback moments but also of feedback messages that are iterative: the feedback that respondents receive later on during the intervention should not only concern the respondent’s present state, but also refer to the changes respondents have made since their enrollment in the program.

The Internet has been discovered to be a popular gateway for delivering health behavior change interventions in general [14] and computer-tailored and smoking cessation interventions in particular [15,16]. Using the Internet to provide such programs may have several advantages for both provider and receiver: it is highly accessible [17,18], it has the potential to reach a large audience at minimal cost, and participants can take part at any time that is most convenient to them. Furthermore, the behavioral feedback given in computer-tailoring programs can still be highly individualized and personal [19]. Moreover, smokers might not succeed the first time they try to quit [20], and seeking help online relatively anonymously may prevent them from feelings of failure and embarrassment, negative feelings that have been shown to be related to a higher temptation to smoke [21] and poorer abstinence outcomes [22].

Although a key element of computer tailoring is that the intervention materials are adapted to specific respondent characteristics, some smokers might benefit more than others from particular smoking cessation interventions. For example, the level of nicotine dependence has previously been suggested to moderate the effectiveness of smoking cessation interventions [23]. Moreover, a study among nicotine patch users identified several participant characteristics moderating the effectiveness of a Web-based computer-tailored intervention [24]. It has therefore been recommended, especially with computer-tailored interventions using new media technologies such as the Internet, to investigate which participant characteristics are associated with effectiveness [4].

As Web-based multiple tailored smoking cessation feedback has not yet been offered to the Dutch general public outside scientific studies, our research group developed a Web-based multiple computer-tailored smoking cessation program and offered Dutch adult smokers the opportunity to participate in this program. The present study investigated the effectiveness of this program on smoking cessation outcomes reported after 6 weeks and 6 months. To imitate a natural situation in which smokers who do not participate in a smoking cessation program do not receive the intervention, the control group did not receive any of the intervention’s components. Nevertheless, both the intervention and control group were free to use other smoking cessation aids during the study period. In addition, we investigated whether the effect of the intervention was different for specific subgroups of smokers and whether we could detect a dose–response relationship between the number of feedback messages received and abstinence at the last follow-up.

## Methods

### Intervention

**Figure 1:**
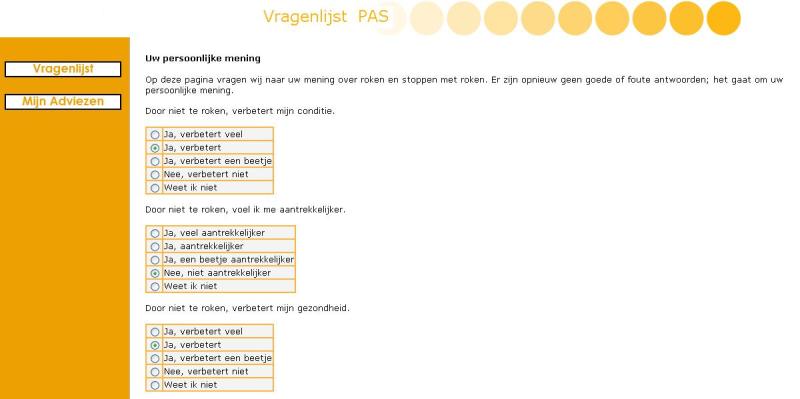
Screenshot of items regarding the pros of smoking cessation.

**Figure 2:**
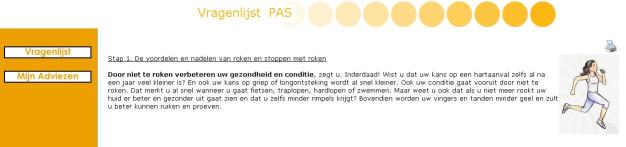
Screenshot of personal advice regarding the pros of smoking cessation.

The Web-based multiple computer-tailored smoking cessation program was based on a previously developed effective single computer-tailored intervention [6], while the I-Change model served as the theoretical framework [25]. While filling out the online baseline questionnaire, all respondents were asked to set a quit date within the next 4 weeks. Respondents in both intervention arms were prompted by email to fill in an online follow-up questionnaire 2 days after their set quit date, and after 6 weeks and 6 months. By clicking on a link provided in this email, respondents could start filling out their next follow-up questionnaire immediately, by logging into the system. One email reminder was sent each time that, 1 week after receiving the first invitation, a respondent had still not filled out the particular questionnaire he or she was invited to complete. While respondents in the control group filled out only the questions, for those in the intervention group questions were directly succeeded by relevant online feedback in order to maintain the respondent’s attention and to improve retention rates. Iterative and item-based feedback messages were tailored to several respondent characteristics [25]: gender, cognitive variables (attitude, social influence, and self-efficacy), intention to quit smoking, goal and relapse prevention strategies (action and coping plans), and smoking behavior. When the questionnaire was completed, feedback messages were combined into one personalized feedback letter. In addition to being able to read the feedback letters on the computer screen, respondents were also sent the feedback letters by email, which allowed for the letters to be printed. The 4- to 5-page feedback letters respondents received at baseline and after 6 weeks consisted of seven components: (1) introduction, including specific feedback on the respondent’s smoking behavior and on his or her intention to quit smoking or to maintain nonsmoking, (2) feedback on the respondent’s attitude (perceived advantages [pros] and disadvantages [cons]) toward smoking and quitting smoking, (3) feedback on perceived social influence (not) to smoke, (4) feedback on the respondent’s reported self-efficacy to refrain from smoking in specific situations, including suggestions on how to cope with these situations, (5) feedback on the extent to which respondents were planning to undertake specific actions (action plans) while preparing their quit attempt, (6) feedback on how to cope with certain difficult situations (coping plans), including the formulation of personal plans in the shape of if–then statements [26], and (7) ending. As we wanted to minimize the burden of filling out a questionnaire by smokers who had recently quit, the feedback letter that respondents received 2 days after the set quit date consisted of only 1 page, giving feedback on smoking (cessation) behavior and relapse prevention strategies. Figure 1 shows an example of exemplary items regarding the pros of smoking cessation. Figure 2 shows an example of a tailored feedback message.

 All respondents in the experimental condition received at least one tailored feedback letter (ie, at baseline). At the 6-week follow-up, respondents could have received at most two tailored feedback letters (ie, at baseline and 2 days after their set quit date), and at the 6-month follow-up, they could have received a maximum of three tailored feedback letters (ie, at baseline, 2 days after their set quit date, and at the 6-week follow-up).

### Recruitment and Procedure

This study was approved by the Medical Ethics Committee of Maastricht University and the University Hospital Maastricht (MEC 08-3-037; NL22692.068.08), and is registered with the Dutch Trial Register (NTR1351). A full description of the study protocol is provided elsewhere [27].

We recruited adult smokers from December 2009 to June 2010 by advertising our study in the mass media and on the Internet. We sent several press releases to regional newspapers in the Netherlands. Most of these newspapers subsequently mentioned our study on their website, included an item about the project in the print version of their newspaper, or mentioned our study on their local radio station or television channel, or both. We also used a Dutch online social network website (Hyves) and several online smoking cessation forums to disseminate our recruitment text. In addition, we advertised our study in a free national newspaper obtainable at all Dutch train stations and several other public places throughout the Netherlands.

After 12 months, we expected a 10% point prevalence abstinence rate in the control condition. Based on results from previous projects, we expected the multiple tailoring program to lead to a 20% point prevalence abstinence rate. To be able to detect this difference significantly (alpha = 5%, beta = 10%), according to a 2-tailed Fisher exact test, 281 respondents per arm were required at the end of the trial (562 respondents in total) [28]. Allowing for 50% attrition over the trial period, 1124 respondents needed to be included at baseline.

Interested smokers could sign up for the study on the study website (http://www.persoonlijkstopadvies.nl) and were eligible to participate if they were 18 years of age or older, were motivated to quit smoking within 6 months, and had access to the Internet. On the study website, participants were informed that the study was financed by the Dutch Cancer Society and conducted by researchers from Maastricht University in cooperation with the Dutch Expert Center on Tobacco Control (STIVORO). Additionally, the website included information about the objectives of the study, the randomization procedure and the incentive provided when respondents completed all questionnaires (ie, a €10 voucher). Respondents could choose their own username and password and were informed that no one but the research team was able to retrieve these passwords. As respondents had to report their email address when signing up for the study, we could easily flag respondents with multiple identities and remove them from further analyses. After providing online informed consent, participants were randomly assigned to the intervention group or the control group by a computer software randomization device, allocating approximately 50% of all respondents to each group. Blinding of respondents was not possible, as they had to take notice of whether they were receiving tailored feedback.

### Measurements

All questionnaires used in the present study were previously used and tested among Dutch smoking adults and were self-administered online [6,28,29].

#### Baseline Measurement

We measured six demographic variables: age, gender (1 = male, 2 = female), educational level (1 = low: primary school/basic vocational school, 2 = medium: secondary vocational school/high school degree, 3 = high: higher vocational school/college degree/university degree), nationality (1 = Dutch, 2 = non-Dutch), and the occurrence of cardiovascular and respiratory diseases (1 = no, 2 = yes).

Exclusion criteria were based on current smoking behavior and motivation to quit smoking: current smoking behavior was measured by 1 item asking whether the respondent had smoked during the past 7 days (1 = no, 2 = yes). Motivation to quit smoking was measured by an adapted version of the Stage of Change algorithm [30]. We asked respondents to state within what time span they intended to quit smoking (1 = not within 6 months, 2 = within 6 months, 3 = within 1 month, 4 = I have quit, but no longer than 6 months, 5 = I have quit for longer than 6 months). Respondents who indicated that they had not smoked during the past 7 days, who were not willing to quit within 6 months, or who had quit already were excluded from further participation.

We measured overall tobacco consumption using five open-ended questions regarding the number of cigarettes, hand-rolled cigarettes, cigars, cigarillos, and pipes smoked per day. Subsequently, the answers on these five questions were converted into an overall score for tobacco consumption (expressed as number of cigarettes), whereby 1 hand-rolled cigarette or cigarillo equaled 1 cigarette and 1 cigar equaled 4 cigarettes [31]. As no concrete guidelines were available for the number of cigarettes that would equal 1 pipe, we conservatively considered 1 pipe to equal 1 cigarette.

We measured addiction level by the abbreviated Fagerström Test for Nicotine Dependence (0 = not addicted, 10 = highly addicted) [32].

We assessed the number of past quit attempts with 1 item, asking the respondents how often they had tried to quit smoking in the past.

#### Follow-up Measurement

At the 6-week and 6-month follow-ups, we assessed prolonged abstinence by 1 item asking whether the respondent had refrained from smoking since the previous measurement (1 = no, 2 = yes). At the 6-week follow-up, prolonged abstinence referred to abstinence since the questionnaire that respondents received 2 days after their set quit date (ie, at least 2 weeks of abstinence). At the 6-month follow-up, this measure referred to abstinence since the 6-week follow-up (ie, 4.5 months of abstinence). In addition, at both follow-ups we assessed 24-hour and 7-day point prevalence abstinence, each by 1 item asking whether the respondent had refrained from smoking during the past 24 hours or 7 days (1 = no, 2 = yes).

### Statistical Analyses

First, we conducted descriptive analyses to determine the sample’s characteristics. To check for differences between the intervention and control groups, we conducted 2-sided *t *tests and chi-square tests. Additionally, to determine whether selective dropout had occurred, we compared those remaining in the study versus those lost to follow-up after 6 weeks and 6 months using 2-sided *t *tests and chi-square tests.

Second, we conducted logistic regression analyses to determine whether the intervention had an effect on the outcome measures assessed after follow-up periods of 6 weeks and 6 months. A negative scenario was used to replace missing values. That is, respondents lost to follow-up were considered to still be smoking. To test the robustness of the results, these analyses were also conducted with complete cases only.

Third, to determine whether the effect of the intervention was different for specific subgroups of smokers, we investigated whether we could identify interaction effects between the study condition and baseline demographic or behavioral measures using logistic regression analyses.

Data were analyzed using SPSS 17.0 (IBM Corporation, Somers, NY, USA). The significance level used was *P *< .05.

## Results

### Sample Characteristics

**Figure 3:**
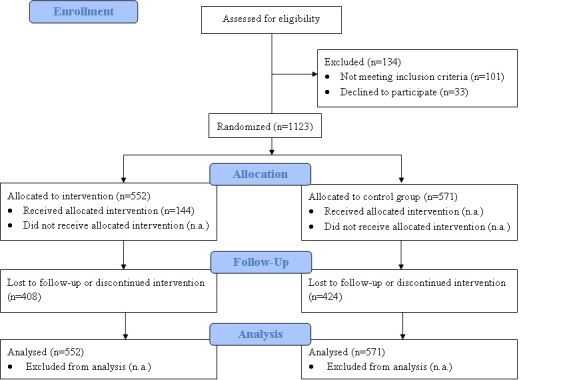
Flow of respondents from enrollment in the study to allocation to the experimental and control conditions, retention, and whether they were included in the analysis.

Figure 3 shows the flow of respondents from enrollment in the study to allocation to the experimental and control conditions, retention, and whether they were included in the analysis. Of the 1257 respondents assessed for eligibility, 33 (3%) declined to participate, 32 (3%) were nonsmokers at baseline, and 69 (6%) were not motivated to quit within 6 months. Ultimately, 1123 (89.34%) respondents were randomly assigned to either the experimental (n = 552) or control (n = 571) group and completed the baseline questionnaire. Of the 1123 respondents included, 449 (40.0%) completed the 6-week follow-up questionnaire and 291 (25.9%) completed the questionnaire at the 6-month follow-up.

Respondents included in the analyses had a mean age of 49.5 years; 535 (47.6%) were male; and 513 (45.7%) had a medium level of education. Respondents in the experimental group significantly differed from those in the control condition in their level of education (χ^2^_2 _= 6.11, *P *= .047). Therefore, educational level was included in subsequent analyses as a potential confounder. As no data concerning level of education were missing for any of the respondents, we included all 1123 in further analyses. Table 1 shows the baseline characteristics of the overall sample and of the experimental and control groups separately.

**Table 1:** Baseline sample characteristics of Dutch smoking adults (N = 1123) recruited from December 2009 to June 2010.

Characteristic		Overall sample (N = 1123)	Experimental group (n = 552)	Control group (n = 571)
Age (years), mean (SD)		49.5 (32.5)	48.4 (12.2)	48.8 (12.3)
Male, % (n)		47.6% (535)	45.8% (253)	49.4% (282)
**Educational level, % (n)**				
	High	21.2% (238)	19.6% (108)	22.8% (130)
	Medium	45.7% (513)	43.8% (242)	47.5% (271)
	Low	33.1% (372)	36.6% (202)	29.8% (170)
Dutch, % (n)		97.7% (1097)	97.8% (540)	97.5% (557)
With cardiovascular diseases, % (n)		9.4% (106)	11.1% (61)	7.9% (45)
With respiratory diseases, % (n)		14.3% (161)	12.5% (69)	16.1% (92)
Number of cigarettes smoked/day, mean (SD)		20.6 (12.4)	20.8 (13.7)	20.4 (11.0)
FTND^a ^score (range 1–10), mean (SD)		5.1 (2.5)	5.0 (2.5)	5.2 (2.4)
Number of previous quit attempts, mean (SD)		5.4 (17.5)	5.1 (10.1)	5.7 (22.4)

^a ^Fagerström Test for Nicotine Dependence.

As Table 2 shows, no differences were found with regard to baseline characteristics between respondents followed up and respondents lost to follow-up after a 6-week period. After 6 months, however, respondents lost to follow-up were significantly younger (*P *= .02) and significantly more addicted (*P *= .01) than those who remained in the study.

**Table 2:** Comparison between respondents followed up and respondents lost to follow-up after 6 weeks and 6 months.

Characteristic		6-week follow-up		6-month follow-up	
		Followed up (n = 449)	Lost to follow-up (n = 674)	Followed up (n = 291)	Lost to follow-up (n = 832)
Age (years), mean (SD)		50.1 (12.2)	49.4 (12.6)	50.0 (12.2)*	48.1 (12.3)*
Male, % (n)		44.5% (200)	49.7% (335)	45.5% (133)	48.4% (402)
In experimental condition, % (n)		49.9% (224)	48.7% (328)	49.3% (144)	49.1% (408)
**Educational level, % (n)**					
	High	19% (85)	22.7% (153)	19% (54)	22.1% (184)
	Medium	45.9% (206)	45.5% (307)	45.2% (132)	45.8% (381)
	Low	35.2% (158)	31.8% (214)	36.3% (106)	32.0% (266)
Dutch, % (n)		98.2% (441)	97.3% (656)	97.3% (284)	97.8% (813)
With cardiovascular diseases, % (n)		12% (52)	8% (54)	11% (31)	9% (75)
With respiratory diseases, % (n)		15% (68)	14% (93)	17% (49)	13.5% (112)
Number of cigarettes smoked/day, mean (SD)		19.8 (12.1)	17.8 (6.1)	19.5 (11.4)	21.0 (12.7)
FTND^a ^score (range 1–10), mean (SD)		4.8 (2.3)	4.6 (2.3)	4.7 (2.3)*	5.2 (2.5)*
Number of previous quit attempts, mean (SD)		5.0 (10.6)	5.5 (5.9)	5.1 (10.0)	5.6 (19.5)

^a ^Fagerström Test for Nicotine Dependence.

*P < .05.

### Effect of the Intervention on Abstinence

Of the 552 respondents in the intervention group, 91 (17%) reported that they had refrained from smoking during the past 24 hours, 74 (13%) reported that they had not smoked during the past 7 days, and 60 (11%) reported that they had not smoked since the previous measurement 2 days after their quit date. In the control group (n = 571) these numbers were 55 (10%), 38 (7%), and 33 (6%), respectively. The intervention had a significant effect on all outcome measures, even when controlling for the baseline difference between the intervention and control groups with regard to their level of education (Table 3). Significantly more respondents in the intervention group than in the control group reported having been abstinent for the past 24 hours, the past 7 days, or since the previous measurement. Results from complete-case analyses were similar (Multimedia Appendix 1).

After 6 months, a total of 51 (9%) respondents in the intervention group reported having refrained from smoking during the past 24 hours, 45 (8%) reported not having smoked during the past 7 days, and 23 (4%) reported not having smoked since the previous measurement. In the control group these numbers were 36 (6%), 34 (6%), and 19 (3%), respectively. Table 4 shows that no significant intervention effects were found with regard to all outcome measures reported at the 6-month follow-up. The complete-case analyses yielded similar results, though they were slightly more positive regarding 24-hour point prevalence abstinence (Multimedia Appendix 1).

We investigated interaction effects between condition and baseline demographic or behavioral measures, although none of these turned out to have a significant influence on any of the abstinence measures reported after 6 weeks or 6 months (data not reported).

**Table 3:** Effects of the Web-based smoking cessation intervention on several behavioral outcomes at 6-week follow-up among Dutch adult smokers (N = 1123) recruited from December 2009 to June 2010.

Model		24-hour ppa^a^			7-day ppa			Prolonged abstinence		
		OR^b^	95% CI^c^	*P *value	OR	95% CI	*P *value	OR	95% CI	*P *value
Intervention^d^		1.85	1.30–2.65	.001*	2.17	1.44–3.27	<.001*	1.99	1.28–3.09	.002*

**Intervention** ^d^		1.81	1.26–2.59	.001*	2.16	1.43–3.25	<.001*	1.96	1.26–3.05	.003*
	Medium education^e^	0.81	0.51–1.32	.42	0.75	0.45–1.36	.28	0.75	0.41–1.32	.31
	High education^e^	1.29	0.81–2.08	.29	0.97	0.58–1.64	.91	1.08	0.61–1.90	.80

^a ^Point prevalence abstinence.

^b ^Odds ratio.

^c ^Confidence interval.

^d ^Control group is the reference category.

^e ^Low education is the reference category.

*P < .05.

**Table 4:** Effects of the Web-based smoking cessation intervention on several behavioral outcomes at 6-month follow-up among Dutch adult smokers (N = 1123) recruited from December 2009 to June 2010.

Model		24-hour ppa^a^			7-day ppa			Prolonged abstinence		
		OR^b^	95% CI^c^	*P *value	OR	95% CI	*P *value	OR	95% CI	*P *value
Intervention^d^		1.51	0.97–2.35	.07	1.40	0.88–2.22	.16	1.26	0.68–2.34	.46

**Intervention** ^d^		1.47	0.94–2.30	.09	1.38	0.87–2.20	.17	1.29	0.69–2.41	.42
	Medium education^e^	0.88	0.48–1.62	.69	0.86	0.47–1.58	.62	0.59	0.28–1.24	.16
	High education^e^	1.38	0.76–2.52	.29	1.10	0.59–2.05	.76	0.56	0.25–1.26	.16

^a ^Point prevalence abstinence.

^b ^Odds ratio.

^c ^Confidence interval.

^d ^Control group is the reference category.

^e ^Low education is the reference category.

## Discussion

### Main Findings

In the present study we investigated the effects of a multiple computer-tailored smoking cessation program delivered through the Internet. The results presented suggest significant effects of the intervention on short-term abstinence: at the 6-week follow-up, respondents who received the intervention were more likely to report being abstinent for the past 24 hours, for the past 7 days, and since the previous measurement (ie, 2 days after their quit date) than those who did not receive the intervention. Despite incorporating goal and relapse prevention strategies (action and coping plans), however, we found no effect of the intervention on abstinence measures assessed after 6 months.

A potential explanation for not finding any suggestion of intervention effects on long-term abstinence might be that more than 70% of the values on the primary outcome measures had to be replaced, as our study had relatively high levels of attrition, as have many previously developed Web-based interventions [16,33-37]. In line with the Russell standard [37], we chose a negative scenario to replace missing values (ie, we considered respondents lost to follow-up to still be smoking). Although this is a recommended analysis when having to deal with dropout [37], replacing more than 70% of the values on the primary outcome measures might increase the chances of making a type II error [34,38]. This may have resulted in an underestimation of the intervention’s effectiveness for those who continued to use it [34]. On the other hand, analyses with complete cases only are likely to increase the chances of a type I error, which would have resulted in unjustified conclusions in favor of the intervention studied. Although in our study the results from both types of analyses were congruent with each other, the possibilities of errors should be kept in mind when interpreting the results presented. To prevent these problems from occurring in the first place, it is of utmost importance to identify strategies that will ensure the sustained use of Web-based interventions. Previously, several suggestions have been made to prevent attrition, such as ensuring high levels of motivation to quit, providing prompts or reminders, preventing self-control depletion, for example, by having respondents form implementation intentions [36] and providing incentives of at least €10 [39]. In addition, a recent review showed that interventions that combined several of these strategies were most effective at facilitating exposure to Web-based interventions [40]. While we took all of the strategies mentioned into account, attrition rates in this study remained high. Evidently, more research is needed to identify strategies that will prevent smokers from dropping out of Web-based behavior change interventions. Qualitative research among respondents lost to follow-up might further illuminate the main reasons why these respondents discontinued a Web-based intervention. In a recent study conducted among problem drinkers, the most common reasons for not completing a Web-based intervention were personal reasons unrelated to the Web-based intervention, followed by dissatisfaction with the intervention and satisfaction with the improvement in their condition [41]. Based on the identification of the reasons for discontinuation, novel strategies to prevent attrition need to be developed and tested. In addition, more research is needed to identify strategies for dealing with missing data due to high attrition rates. A recent study comparing six different approaches to missing data concluded that multiple imputation might yield the most valid results [33]. However, as the assumption that respondents who drop out should be considered to still be smoking is well established and a still-recommended strategy in smoking cessation research [37], in the present study we opted for this strategy.

Another possible explanation for the lack of intervention effects on long-term abstinence may be that Web-based smoking cessation programs are not sufficiently tailored and adapted to the long-term wishes of recent ex-smokers to prevent relapse to smoking. Respondents received feedback only at fixed points in time; it was not possible to obtain additional personal feedback or support at times when smokers might have needed it most. The integration of ecological momentary assessment, by collecting real-time data through, for example, palmtops, personal digital assistants, or electronic diaries, might be promising. Studies using palmtop computers showed that a decrease in self-efficacy, an increase in positive smoking outcome expectancies, and an increase in negative affect predicted the occurrence of a lapse to smoking on the next day [42,43]. Integrating ecological momentary assessment into a Web-based intervention might enable us to monitor fluctuations in factors such as self-efficacy and negative affect and, as a consequence, enable us to adapt intervention materials to the needs of recent ex-smokers and, ultimately, to prevent lapses and relapse. In addition, the finding that those lost to follow-up were significantly more addicted to nicotine than those who remained in the study supports the idea that insufficient attention was paid to dealing with withdrawal symptoms. Although, in line with current guidelines [44], we advised smokers who reported smoking more than 10 cigarettes per day to use smoking cessation medication, we did not assess whether these smokers did in fact use such medication during their quit attempt. Even though the Web-based intervention provided information on physical withdrawal symptoms and how to deal with these symptoms, all feedback messages targeted cognitions. As a consequence, solely reading these messages might not have decreased physical withdrawal symptoms. As addiction has been shown to be the most important predictor of a quit attempt’s success [45,46], it may be possible to obtain higher success rates when Web-based smoking cessation interventions are combined with smoking cessation medication aimed at reducing physical withdrawal symptoms. Varenicline, for instance, has been shown to attenuate physical withdrawal symptoms and to prevent relapse to smoking [47,48].

We found no support for different intervention effects for specific subgroups of smokers. Based on the results, it could thus be argued that the intervention was equally effective for all smokers who participated in the program. However, respondents who dropped out of the study were relatively more addicted and relatively younger than those who remained in the study, which is in line with previous research [49,50]. A potential explanation might be that younger people have not yet experienced any smoking-related health effects and are, compared with older people who are more often confronted with chronic diseases, less internally motivated to invest time in health behavior change interventions [51].

### Study Strengths and Limitations

Major strengths of the present study were the large sample of smokers who initiated participation in the smoking cessation program and the relatively long follow-up period. However, as mentioned previously, the study had relatively high dropout rates. In the present study, we applied several strategies previously suggested to prevent attrition [36,39]: using motivation to quit as an inclusion criterion, sending two reminder emails for each follow-up questionnaire, encouraging respondents to formulate coping plans in the form of implementation intentions, and providing respondents with a €10 voucher for completing all follow-up questionnaires. Despite the actions taken, however, attrition rates remained high. A second limitation is that we could conduct no appropriate dose–response analysis. Insufficient data were available for participants who received one, two, or three letters and who also provided 6-month follow-up data. Of the respondents in the intervention group who provided 6-month follow-up data (n = 144), almost 80% (n = 115) received the highest dose of three feedback letters, which resulted in insufficient variation in the doses received to conduct this analysis. As previously stated, we found a significant dose–response relationship between the number of feedback moments and smoking abstinence [13]. We therefore recommend that future studies conduct a dose–response analysis to determine whether this effect can be replicated. Finally, we were unable to use continued abstinence as an outcome measure, as all respondents were asked to set a quit date within 4 weeks from filling out the baseline questionnaire and were not obliged to quit immediately. According to the Russell standard, however, continued abstinence may classify too many successes as failures due to its strict criteria [37]. As we used prolonged abstinence instead, we were still able to assess a long period of abstinence (ie, at least 4.5 months).

### Conclusions

This Web-based computer-tailored smoking cessation program had a significant effect on abstinence measured after a 6-week follow-up period. However, this effect had entirely disappeared after 6 months. To prevent relapse, future studies should focus on the possibility of applying an ecological momentary assessment or combining the present Web-based intervention with the use of smoking cessation medication. Moreover, further research should aim at identifying strategies to prevent smokers from dropping out of Web-based smoking cessation interventions. As complete-case analyses and the replacement of missing values using a negative scenario both have their limitations, alternative strategies should be identified and tested.

## Multimedia Appendix 1

Complete-case analyses.

## Multimedia Appendix 2

CONSORT-EHEALTH Checklist (V1.6) [52].

## Abbreviations

CI: confidence interval
OR: odds ratio
