# Supplementary material for: Effectiveness of a Web-Based Multiple Tailored Smoking Cessation Program: A Randomized Controlled Trial Among Dutch Adult Smokers
Source: J Med Internet Res. 2012 Jun 11;14(3):e82. doi: 10.2196/jmir.1812 (PMC3414857; doi:10.2196/jmir.1812)
Supplement: Supplementary file 1 [file jmir_v14i3e82_app1.pdf]

APPENDIX 1 Results from complete-case analyses

Table 5. Effects of the web-based smoking cessation intervention on several behavioural outcomes at six-week follow-up among Dutch adult smokers (N=449) recruited from December 2009 up until June 2010 - complete cases

Model	24-hour ppa[a]			7-day ppa[a]			Prolonged abstinence		
	OR[b]	95% CI[c]	Pvalue	OR[b]	95% CI[c]	Pvalue	OR[b]	95% CI[c]	Pvalue
Intervention[d]	2.12	1.41-3.17	.000*	2.43	1.55-3.79	.000*	2.13	1.33-3.42	.002*
Intervention[d]	2.07	1.38-3.13	.001*	2.50	1.59-3.94	.000*	2.14	1.32-3.46	.002*
Medium education[e]	.63	.36-1.09	.10	.57	.32-1.02	.06	.59	.31-1.09	.09
High education[e]	1.00	.57-1.76	1.00	.68	.37-1.24	.21	.80	.43-1.51	.49

[a]Point prevalence abstinence.
[b] Odds ratio.
[c] Confidence interval.
[d] Control group is the reference category.
[e] Low education is the reference category.
**P* < .05.

Table 6. Effects of the web-based smoking cessation intervention on several behavioural outcomes at six-month follow-up among Dutch adult smokers (N=291) recruited from December 2009 up until June 2010 - complete cases

Model	24-hour ppa[a]			7-day ppa[a]			Prolonged abstinence		
	OR[b]	95% CI[c]	Pvalue	OR[b]	95% CI[c]	Pvalue	OR[b]	95% CI[c]	Pvalue
Intervention[d]	1.69	1.02-2.81	.04*	1.51	.90-2.54	.12	1.29	.67-2.49	.45
Intervention[d]	1.65	.98-2.76	.06	1.54	.90-2.61	.11	1.46	.74-2.87	.27
Medium education[e]	.68	.33-1.37	.28	.65	.32-1.32	.24	.45	.20-1.01	.05
High education[e]	1.02	.50-2.08	.97	.76	.37-1.57	.45	.37	.15-.89	.03*

[a]Point prevalence abstinence.

[b] Odds ratio.
[c] Confidence interval.
[d] Control group is the reference category.
[e] Low education is the reference category.
*$P < .05$.
