# Supplementary material for: Effectiveness of a Web-Based Multiple Tailored Smoking Cessation Program: A Randomized Controlled Trial Among Dutch Adult Smokers
Source: J Med Internet Res. 2012 Jun 11;14(3):e82. doi: 10.2196/jmir.1812 (PMC3414857; doi:10.2196/jmir.1812)
Supplement: Supplementary file 2 [file jmir_v14i3e82_app2.pdf]

CONSORT-EHEALTH Checklist V1.6 Report	Manuscript Number	1812
Date completed	8/25/2011 14:50:29	
by	Eline Suzanne Smit	
Effectiveness of a web-based multiple tailored smoking cessation program: a randomized controlled trial among Dutch adult smokers		
TITLE		
**1a-i) Identify the mode of delivery in the title**		
"Effectiveness of a web-based multiple tailored smoking cessation program: a randomized controlled trial among Dutch adult smokers"		
**1a-ii) Non-web-based components or important co-interventions in title**		
n.a.		
**1a-iii) Primary condition or target group in the title**		
"Effectiveness of a web-based multiple tailored smoking cessation program: a randomized controlled trial among Dutch adult smokers"		
ABSTRACT		
**1b-i) Key features/functionalities/components of the intervention and comparator in the METHODS section of the ABSTRACT**		
"Respondents in the experimental group received the fully automated web based smoking cessation program, while respondents in the control group received no intervention."		
**1b-ii) Level of human involvement in the METHODS section of the ABSTRACT**		
"Respondents in the experimental group received the fully automated web based smoking cessation program, while respondents in the control group received no intervention."		
**1b-iii) Open vs. closed, web-based (self-assessment) vs. face-to-face assessments in the METHODS section of the ABSTRACT**		
"Smokers were recruited from December 2009 to June 2010 by advertising our study in the mass media and on the Internet. Those interested and motivated to quit smoking within six months (N=1123) were randomized into either the experimental (N=552) or control group (N=571). After six weeks and after six months, the effect of the intervention on self-reported 24 hour point prevalence abstinence, 7 day point prevalence abstinence and prolonged abstinence was assessed using logistic regression analyses."		
**1b-iv) RESULTS section in abstract must contain use data**		
"Of the 1123 respondents included, 449 (40.0%) completed the six week follow-up questionnaire, while 291 (25.9%) completed the questionnaire at six-month follow-up. The computer-tailored program significantly increased 24 hour point prevalence abstinence (OR 1.85; 95% CI 1.30-2.65), 7 day point prevalence abstinence (OR 2.17; 95% CI 1.44-3.27) and prolonged abstinence (OR 1.99; 95% CI 1.28-3.09) rates reported after six weeks. After six months, an effect of the intervention was found on 24-hour point prevalence abstinence that approached significance (p=.07) (OR 1.51; 95% CI .97-2.35), while no intervention effect was found on 7-day point prevalence and prolonged abstinence."		
**1b-v) CONCLUSIONS/DISCUSSION in abstract for negative trials**		
"The web-based computer-tailored smoking cessation program had a significant effect on abstinence reported after a six-week period. Nonetheless, this effect totally disappeared at six-month follow-up. To prevent relapse, future studies should focus on the possibility of applying ecological momentary assessment or combining the present web-based intervention with the use of smoking cessation medication, such as varenicline. Moreover, further research should aim at identifying the best strategies for dealing with missing values and at the identification of strategies that will prevent smokers from dropping out of web-based smoking cessation interventions and, as complete-case analyses and the replacement of missing values using a negative scenario both have their limitations, alternative strategies should be identified and tested."		
INTRODUCTION		
**2a-i) Problem and the type of system/solution**		
"One strategy that has shown both short and long term efficacy in changing smoking behaviour is computer tailoring. A computer tailoring approach may be warranted that not only consists of multiple feedback moments but also of feedback messages that are iterative. Using the Internet to provide such programs may have several advantages for both provider and receiver."		
**2a-ii) Scientific background, rationale: What is known about the (type of) system**		

"As web-based multiple tailored smoking cessation feedback has not yet been offered to the Dutch general public outside scientific studies, our research group developed a web based multiple computer tailored smoking cessation program and offered Dutch adult smokers the opportunity to participate in this program. The present study investigated the effectiveness of this program on smoking cessation outcomes reported after six weeks and six months. To imitate a natural situation in which smokers who do not participate in a smoking cessation program do not receive the intervention, the control group did not receive any of the intervention's components. Nevertheless, both the intervention and control group were free to use other smoking cessation aids during the study period. In addition, we investigated whether the effect of the intervention was different for specific subgroups of smokers and whether a dose-response relationship could be detected between the number of feedback messages received and abstinence at the last follow-up."		
METHODS		
**3a) CONSORT**		
"The present study investigated the effectiveness of this program on smoking cessation outcomes reported after six weeks and six months."<br>"In addition, we investigated whether the effect of the intervention was different for specific subgroups of smokers and whether a dose-response relationship could be detected between the number of feedback messages received and abstinence at the last follow-up."		
**3b-i) Bug fixes, Downtimes, Content Changes**		
n.a.		
**4a-i) Computer / Internet literacy**		
"Interested smokers could sign up for the study on the study website (http://www.persoonlijkstopadvies.nl) and were eligible to participate when they were 18 years or older, were motivated to quit smoking within six months and had access to the Internet."		
**4a-ii) Open vs. closed, web-based vs. face-to-face assessments:**		
"Adult smokers were recruited from December 2009 until June 2010 by advertising our study in the mass media and on the Internet. We also used a Dutch online social network website (Hyves), several online quit smoking cessation forums and we advertised our study in a free national newspaper. After providing online informed consent, participants were randomized into the intervention group or the control group using a computer software randomization device, allocating approximately 50% of all respondents to both groups. As respondents had to report their e-mail address when signing up for the study, respondents with multiple identities were easily identified."		
**4a-iii) Information giving during recruitment**		
"Additionally, respondents were informed about the objectives of the study, the randomization procedure and the incentive provided when they completed all questionnaires, i.e. a 10 euro voucher. Respondents could sign up for the study with their own username and password and were informed that no one but the PAS research team was able to retrieve these passwords. After providing online informed consent, …"		
**4b-i) Report if outcomes were (self-)assessed through online questionnaires**		
"All questionnaires … were self-administered online."		
**4b-ii) Report how institutional affiliations are displayed**		
"On the study website, participants were informed that the study was financed by the Dutch Cancer Society and conducted by researchers from Maastricht University in cooperation with the Dutch Expert Center on Tobacco Control (STIVORO)."		
**5-i) Mention names, credential, affiliations of the developers, sponsors, and owners**		
"The web-based multiple computer-tailored smoking cessation program was developed by the study team, based on a previously developed effective single computer-tailored intervention [6]. The I-Change model served as the theoretical framework [25]. The software used to build the program has been developed and is owned by OverNite Software Europe B.V."		
**5-ii) Describe the history/development process**		
"The web-based multiple computer-tailored smoking cessation program was based on a previously developed effective single computer-tailored intervention, while the I-Change model served as the theoretical framework."		
**5-iii) Revisions and updating**		
n.a.		
**5-iv) Quality assurance methods**		
n.a.		
**5-v) Ensure replicability by publishing the source code, and/or providing screenshots/screen-capture video, and/or providing flowcharts of the algorithms used**		
"For an example of a tailored feedback message, we refer the reader to another paper."		
**5-vi) Digital preservation**		
"Interested smokers could sign up for the study on the study website (http://www.persoonlijkstopadvies.nl) …"		
**5-vii) Access**		

"While filling out the online baseline questionnaire, all respondents were asked to set a quit date within the next four weeks. Respondents in both intervention arms were prompted by e-mail to fill in an online follow-up questionnaire two days after their set quit date, and after six weeks and six months. By clicking on a link provided in this e-mail, respondents could start filling out their next follow-up questionnaire immediately, by logging in to the system."		
**5-viii) Mode of delivery, features/functionalities/components of the intervention and comparator, and the theoretical framework**		
"The web-based multiple computer-tailored smoking cessation program was based on a previously developed effective single computer-tailored intervention, while the I-Change model served as the theoretical framework. …for those in the intervention group questions were directly succeeded by relevant online feedback in order to maintain the respondent's attention and to improve retention rates. Iterative and item-based feedback messages were tailored to several respondent characteristics: gender, cognitive variables (attitude, social influence and self efficacy), intention to quit smoking, goal and relapse prevention strategies (action and coping plans), and smoking behaviour. When the questionnaire was completed, feedback messages were combined into one personalized feedback letter. In addition to being able to read the feedback letters on the computer screen, respondents were also sent the feedback letters by e-mail, which allowed for the letters to be printed. The feedback letters respondents received at baseline and after six weeks consisted of four to five pages and seven components: 1) introduction, including specific feedback on the respondent's smoking behaviour and on his/her intention to quit smoking or to maintain non-smoking; 2) feedback on the respondent's attitude (perceived advantages (pros) and disadvantages (cons)) about smoking and quitting smoking; 3) feedback on perceived social influence (not) to smoke; 4) feedback on the respondent's reported self-efficacy to refrain from smoking in specific situations, including suggestions on how to cope with these situations; 5) feedback on the extent to which respondents were planning to undertake specific actions (action plans) while preparing their quit attempt; 6) feedback on how to cope with certain difficult situations (coping plans), including the formulation of personal plans in the form of if-then statements; 7) ending. As we wanted to minimize the burden of filling out a questionnaire by smokers who had recently quit, the feedback letter respondents received two days after the set quit date consisted of one page only, giving feedback on (non)smoking behaviour and on relapse prevention strategies. For an example of a tailored feedback message, we refer the reader to another paper."		
**5-ix) Describe use parameters**		
"Respondents in both intervention arms were prompted by e-mail to fill in an online follow-up questionnaire two days after their set quit date, and after six weeks and six months. All respondents in the experimental condition received at least one tailored feedback letter, i.e. at baseline. At six-week follow-up, respondents could at most have received two tailored feedback letters (i.e. at baseline and two days after their set quit date), while at six-month follow-up, they could have received a maximum of three tailored feedback letters (i.e. at baseline, two days after their set quit date and at six-week follow-up)."		
**5-x) Clarify the level of human involvement**		
n.a.		
**5-xi) Report any prompts/reminders used**		
"One e-mail reminder was sent each time that, one week after receiving the first invitation, a respondent had still not filled out the particular questionnaire he or she was invited to complete."		
**5-xii) Describe any co-interventions (incl. training/support)**		
n.a.		
**6a-i) Online questionnaires: describe if they were validated for online use and apply CHERRIES items to describe how the questionnaires were designed/deployed**		
"All questionnaires used in the present study were previously used and tested among Dutch smoking adults and were self-administered online. The description of the questionnaires in the manuscript was checked according to the CHERRIES checklist."		
**6a-ii) Describe whether and how "use" (including intensity of use/dosage) was defined/measured/monitored**		
"All respondents in the experimental condition received at least one tailored feedback letter, i.e. at baseline. At six-week follow-up, respondents could at most have received two tailored feedback letters (i.e. at baseline and two days after their set quit date), while at six-month follow-up, they could have received a maximum of three tailored feedback letters (i.e. at baseline, two days after their set quit date and at six-week follow-up)."		
**6a-iii) Describe whether, how, and when qualitative feedback from participants was obtained**		
n.a.		
**7a-i) Describe whether and how expected attrition was taken into account when calculating the sample size**		
"After twelve months, 10% point prevalence abstinence was expected in the control condition, while based on results from previous projects the multiple tailoring program was expected to lead to 20% point prevalence abstinence. To be able to detect this difference significantly (α=5%; β=10%), according to a two-tailed Fisher's exact test, 281 respondents per arm were required at the end of the trial (562 respondents in total) [27]. Allowing for 50% attrition over the trial period, 1124 respondents needed to be included at baseline."		
**7b) CONSORT**		
n.a.		

**8a) CONSORT**		
"After providing online informed consent, participants were randomized into the intervention group or the control group using a computer software randomization device, allocating approximately 50% of all respondents to both groups. "		
**8b) CONSORT**		
"After providing online informed consent, participants were randomized into the intervention group or the control group using a computer software randomization device, allocating approximately 50% of all respondents to both groups."; n.a.		
**9) CONSORT**		
"After providing online informed consent, participants were randomized into the intervention group or the control group using a computer software randomization device, allocating approximately 50% of all respondents to both groups."		
**10) CONSORT**		
"After providing online informed consent, participants were randomized into the intervention group or the control group using a computer software randomization device, allocating approximately 50% of all respondents to both groups."		
**11a-i) Specify who was blinded, and who wasn't**		
"Blinding of respondents was not possible as they had to take notice of whether or not they were receiving tailored feedback."		
**11a-ii) Discuss e.g., whether participants knew which intervention was the "intervention of interest" and which one was the "comparator"**		
"Additionally, respondents were informed about the objectives of the study, the randomization procedure and the incentive provided when they completed all questionnaires, i.e. a 10 euro voucher. Respondents could sign up for the study with their own username and password and were informed that no one but the PAS research team was able to retrieve these passwords. After providing online informed consent, ... Blinding of respondents was not possible as they had to take notice of whether or not they were receiving tailored feedback."		
**11b) CONSORT**		
n.a.		
**12a) CONSORT**		
"Secondly, logistic regression analyses were conducted to determine whether the intervention had an effect on the outcome measures assessed after follow-up periods of six weeks and six months."		
**12a-i) Imputation techniques to deal with attrition / missing values**		
"A negative scenario was used to replace missing values, i.e. respondents lost to follow-up were considered to still be smoking. To test the robustness of the results, these analyses were also conducted with complete-cases only."		
**12b) CONSORT**		
"Thirdly, to determine whether the effect of the intervention was different for specific subgroups of smokers, we investigated whether interaction effects could be identified between study condition and baseline demographic or behavioural measures using logistic regression analyses."		
RESULTS		
**13a) CONSORT**		
"Figure 1 shows the flow of respondents from enrolment in the study to allocation to the experimental and control condition, retention and whether or not they were included in the analysis. Of the 1257 respondents assessed for eligibility, 33 (3%) declined to participate, 32 (3%) were non-smokers at baseline and 69 (6%) were not motivated to quit within six months. Ultimately, 1123 (89.3%) respondents were randomized into either the experimental (N=552) or control (N=571) group. Of the 1123 respondents included, 449 (40.0%) completed the six week follow-up questionnaire, while 291 (25.9%) completed the questionnaire at six-month follow-up."<br>See also figure 1.		
**13b) CONSORT**		
"Figure 1 shows the flow of respondents from enrolment in the study to allocation to the experimental and control condition, retention and whether or not they were included in the analysis. Of the 1257 respondents assessed for eligibility, 33 (3%) declined to participate, 32 (3%) were non-smokers at baseline and 69 (6%) were not motivated to quit within six months. Ultimately, 1123 (89.3%) respondents were randomized into either the experimental (N=552) or control (N=571) group. Of the 1123 respondents included, 449 (40.0%) completed the six week follow-up questionnaire, while 291 (25.9%) completed the questionnaire at six-month follow-up."<br>See also figure 1.		
**13b-i) Attrition diagram**		
"Figure 1 shows the flow of respondents from enrolment in the study to allocation to the experimental and control condition, retention and whether or not they were included in the analysis. Of the 1257 respondents assessed for eligibility, 33 (3%) declined to participate, 32 (3%) were non-smokers at baseline and 69 (6%) were not motivated to quit within six months. Ultimately, 1123 (89.3%) respondents were randomized into either the experimental (N=552) or control (N=571) group. Of the 1123 respondents included, 449 (40.0%) completed the six week follow-up questionnaire, while 291 (25.9%) completed the questionnaire at six-month follow-up."		

**14a) CONSORT**		
"Adult smokers were recruited from December 2009 until June 2010..."		
**14a-i) Indicate if critical “secular events” fell into the study period**		
n.a.		
**14b) CONSORT**		
n.a.		
**15) CONSORT**		
"Respondents included in the analyses had a mean age of 49.5 years, while 535 (47.6%) of the respondents were male and 513 (45.7%) of them had a medium level of education. Respondents in the experimental group significantly differed from those in the control condition concerning their level of education (χ2=6.11; p=.047). Therefore, educational level was included in subsequent analyses as a potential confounder. Sample characteristics for the overall sample and for the two groups separately can be found in table 1.<br>As is shown in table 2, no differences were found with regard to baseline characteristics between respondents followed up and respondents lost to follow-up after a six week period. After six months, however, respondents lost to follow-up were significantly younger (p=.02) and significantly more addicted (p=.01) than those who remained in the study."<br>See also table 1.		
**15-i) Report demographics associated with digital divide issues**		
"Respondents included in the analyses had a mean age of 49.5 years, while 535 (47.6%) of the respondents were male and 513 (45.7%) of them had a medium level of education. Respondents in the experimental group significantly differed from those in the control condition concerning their level of education (χ2=6.11; p=.047). Therefore, educational level was included in subsequent analyses as a potential confounder. Sample characteristics for the overall sample and for the two groups separately can be found in table 1.<br>As is shown in table 2, no differences were found with regard to baseline characteristics between respondents followed up and respondents lost to follow-up after a six week period. After six months, however, respondents lost to follow-up were significantly younger (p=.02) and significantly more addicted (p=.01) than those who remained in the study."		
**16-i) Report multiple “denominators” and provide definitions**		
"Figure 1 shows the flow of respondents from enrolment in the study to allocation to the experimental and control condition, retention and whether or not they were included in the analysis. Of the 1257 respondents assessed for eligibility, 33 (3%) declined to participate, 32 (3%) were non-smokers at baseline and 69 (6%) were not motivated to quit within six months. Ultimately, 1123 (89.3%) respondents were randomized into either the experimental (N=552) or control (N=571) group. Of the 1123 respondents included, 449 (40.0%) completed the six week follow-up questionnaire, while 291 (25.9%) completed the questionnaire at six-month follow-up."		
**16-ii) Primary analysis should be intent-to-treat**		
"A negative scenario was used to replace missing values, i.e. respondents lost to follow-up were considered to still be smoking. To test the robustness of the results, these analyses were also conducted with complete cases only."		
**17a) CONSORT**		
"Of the 552 respondents in the intervention group, 91 (16%) reported to have refrained from smoking during the past 24 hours, while 74 (13%) reported not to have smoked during the past seven days and 60 (11%) reported not to have smoked since the previous measurement two days after their quit date. In the control group these numbers were 55 (10%), 38 (7%) and 33 (6%), respectively. A significant effect of the intervention was found on all outcome measures, also when controlling for the baseline difference found between the intervention and control group with regard to their level of education (table 3). Respondents in the intervention group significantly more often reported to have been abstinent for the past 24 hours, 7 days or since the previous measurement than respondents in the control group. Results from complete-case analyses were similar (appendix 1).<br>After six months, a total of 51 (9%) respondents in the intervention group reported to have refrained from smoking during the past 24 hours, while 45 (8%) reported not to have smoked during the past seven days and 23 (4%) reported not to have smoked since the previous measurement. In the control group these numbers were 36 (6%), 34 (6%) and 19 (4%), respectively. Table 4 shows that no significant intervention effects were found with regard to all outcome measures reported at six-month follow-up, While complete-case analyses yielded similar results, these results were slightly more positive regarding 24-hour point prevalence abstinence (appendix 1)."<br>See also table 3, table 4 and appendix 1.		
**17a-i) Presentation of process outcomes such as metrics of use and intensity of use**		
"Dose: All respondents in the experimental condition received at least one tailored feedback letter, i.e. at baseline. At six-week follow-up, respondents could at most have received two tailored feedback letters (i.e. at baseline and two days after their set quit date), while at six-month follow-up, they could have received a maximum of three tailored feedback letters (i.e. at baseline, two days after their set quit date and at six-week follow-up)."		
**17b) CONSORT**		

"Of the 552 respondents in the intervention group, 91 (16%) reported to have refrained from smoking during the past 24 hours, while 74 (13%) reported not to have smoked during the past seven days and 60 (11%) reported not to have smoked since the previous measurement two days after their quit date. In the control group these numbers were 55 (10%), 38 (7%) and 33 (6%), respectively. A significant effect of the intervention was found on all outcome measures, also when controlling for the baseline difference found between the intervention and control group with regard to their level of education (table 3). Respondents in the intervention group significantly more often reported to have been abstinent for the past 24 hours, 7 days or since the previous measurement than respondents in the control group. Results from complete-case analyses were similar (appendix 1).<br>After six months, a total of 51 (9%) respondents in the intervention group reported to have refrained from smoking during the past 24 hours, while 45 (8%) reported not to have smoked during the past seven days and 23 (4%) reported not to have smoked since the previous measurement. In the control group these numbers were 36 (6%), 34 (6%) and 19 (4%), respectively. Table 4 shows that no significant intervention effects were found with regard to all outcome measures reported at six-month follow-up, While complete-case analyses yielded similar results, these results were slightly more positive regarding 24-hour point prevalence abstinence (appendix 1)."<br>See also table 3, table 4 and appendix 1.		
**18) CONSORT**		
n.a.		
**18-i) Subgroup analysis of comparing only users**		
n.a.		
**19) CONSORT**		
n.a.		
**19-i) Include privacy breaches, technical problems**		
n.a.		
**19-ii) Include qualitative feedback from participants or observations from staff/researchers**		
n.a.		
DISCUSSION		
**20-i) Typical limitations in ehealth trials**		
"… the study suffered from relatively high drop-out rates.<br><br>A second limitation is that no appropriate dose-response analysis could be conducted. No sufficient data were available of participants who received one, two or three letters and who also provided six-month follow-up data. Of the respondents in the intervention group who provided six-month follow-up data (N=144), almost 80% received the highest dose of three feedback letters, which resulted in not sufficient variation in the doses received to conduct this analysis.<br><br>Thirdly, we were unable to use continued abstinence as an outcome measure as all respondents were asked to set a quit date within four weeks from filling out the baseline questionnaire and were not obliged to quit immediately."		
**21-i) Generalizability to other populations**		
"No support was found for different intervention effects for specific subgroups of smokers. Based on the results presented it could thus be argued that the intervention was equally effective for all smokers who participated in the programme. However, respondents who dropped out of the study were relatively more addicted and relatively younger than those who remained in the study, which is in line with previous research [38, 39]"		
**21-ii) Discuss if there were elements in the RCT that would be different in a routine application setting**		
n.a.		
**22-i) Restate study questions and summarize the answers suggested by the data, starting with primary outcomes and process outcomes (use)**		
"Significant effects of the intervention were found with regard to short-term abstinence: at six-week follow-up, respondents who received the intervention were more likely to report being abstinent for the past 24 hours, the past 7 days and since the previous measurement (i.e. two days after their quit date) than those who did not receive the intervention. Despite the incorporation of goal and relapse prevention strategies (action and coping plans), however, after six months, the effect of the intervention on abstinence measures had completely vanished."		
**22-ii) Highlight unanswered new questions, suggest future research**		

"Moreover, further research should aim at the identification of strategies that will prevent smokers from dropping out of web-based smoking cessation interventions…<br><br>As complete-case analyses and the replacement of missing values using a negative scenario both have their limitations, alternative strategies should be identified and tested.<br><br>Qualitative research among respondents lost to follow-up might be worthwhile to conduct to discover the main reasons for these respondents to discontinue the present intervention.<br><br>… future studies should focus on the possibility of applying ecological momentary assessment or combining the present web-based intervention with the use of smoking cessation medication."		
Other information		
**23) CONSORT**		
"The study towards the intervention's effectiveness was approved by the Medical Ethics Committee of Maastricht University and the University Hospital Maastricht (MEC 08-3-037; NL22692.068.08), and is registered with the Dutch Trial Register (NTR1351). "		
**24) CONSORT**		
"A full description of the study protocol is provided elsewhere [26]. "		
**25) CONSORT**		
"This study was funded by the Dutch Cancer Society (UM 2007-3834)."		
**X26-i) Comment on ethics committee approval**		
"The study towards the intervention's effectiveness was approved by the Medical Ethics Committee of Maastricht University and the University Hospital Maastricht (MEC 08-3-037; NL22692.068.08), and is registered with the Dutch Trial Register (NTR1351)."		
**x26-ii) Outline informed consent procedures**		
"Additionally, respondents were informed about the objectives of the study, the randomization procedure and the incentive provided when they completed all questionnaires, i.e. a 10 euro voucher. Respondents could sign up for the study with their own username and password and were informed that no one but the PAS research team was able to retrieve these passwords. After providing online informed consent, ..."		
**X26-iii) Safety and security procedures**		
"… no one but the PAS research team was able to retrieve these passwords."		
**X27-i) State the relation of the study team towards the system being evaluated**		
"The content of the intervention evaluated in the present study has been developed by the study team."		
